# Bio-catalysis of mango industrial waste by newly isolated *Fusarium* sp. (PSTF1) for pectinase production

**DOI:** 10.1007/s13205-015-0288-3

**Published:** 2015-03-22

**Authors:** M. Purnachandra Reddy, K. V. Saritha

**Affiliations:** 1 Department of Biotechnology, SVU College of Sciences, Sri Venkateswara University, Tirupati, 517 502 Andhra Pradesh India; 2 Department of Future Studies, SVU College of Sciences, Sri Venkateswara University, Tirupati, 517 502 Andhra Pradesh India

**Keywords:** Mango fruit processing industrial waste (MIW), *Fusarium* sp. (PSTF1), Solid state fermentation (SSF), Submerged fermentation (SmF), Pectinase

## Abstract

The dried mango fruit processing industrial waste (MIW) used as carbon source for the production of pectinase from fungal strains. Eight fungal strains were isolated from MIW and screened for their ability to produce pectinase by pectin clear zone (PCZ) technique. *Fusarium* sp. (PSTF1) showed highest PCZ value of 52 mm. The physico-chemical characteristics of the medium were standardized for high production of pectinase. The highest production of pectinase in submerged fermentation observed at temperature—28 °C, pH-6.0, inoculum-size 0.6/25 ml, incubation—72 h, substrate concentration—0.6 g/100 ml, carbon source-fructose (1 %). The effect of different amino acids, vitamins also observed. Under these optimal conditions the highest activity 81.9718 µmol/ml of pectinase was observed. The *Fusarium* sp. (PSTF1) has been considered as the best pectinase producer in submerged fermentation of MIW. The cheap waste raw material used as best carbon source for high production of high value pectinase.

## Introduction

Mango is an important tropical fruit crop of semi-arid countries including India. More than 90 countries all over the world are producing mango fruits. India ranked as top in mango production in the world, with its production of 12,749.8 million tons, in India Andhra Pradesh (AP) stands for second position with its production of 3363.4 million tons. So more number of Mango fruit processing industries were established in AP. During the mango fruit processing 1/3 of fruit goes in the form of waste. Mango fruit processing industries are dumping waste on open fields and usually this waste is used as cattle feed, fuel wood or deposited as landfills (Devendra et al. 2012). This leads to serious environmental pollution, as well as adversely effecting on livestock leading to death due to feeding of this contaminated waste (Andhra Jyothi News Paper, Chittoor, 2012). To overcome this problem MIW is used as a source for the production of pectinase in submerged fermentation. Dried mango industrial waste contains an appreciable amount of pectin, other carbohydrates, proteins, low fat content, and thus can be used as substrate for the production of pectinases from microorganisms. Pectin is the ideal substrate for production of pectinase from mango industrial waste after isolation of potential pectinolytic fungi. Most of the substrates used for solid-state fermentation are materials of plant origin like grains, rice, corn, root, tubers, and legumes. Apart from these, apple pomace, orange waste and other fruit and vegetable industrial wastes and different agro-industrial wastes were used for the production of pectinase, cellulase, α amylase and esterase and peroxidase are also being in much use (Smith and Aidoo 1988; Attyia and Ashour 2002). Pectinase can be produced by both submerged and solid state fermentations by the cultivation of filamentous fungi like *Aspergillus*, *Penicillium*, *Trichoderma*, *Mucor* and *Yarrowia* etc., (Pariza and Foster 1983; Mudgett 1986). Fungi can produce both intracellular as well as extracellular enzymes but for breaking down of larger complex compounds like pectin, fungi secrete extra cellular enzymes (pectinases). The extra cellular enzymes can be easily extracted when compared with intracellular enzymes. Pectinases are group of enzymes that attack pectin (a class of complex polysaccharides found in the cell wall of higher plants and cementing material for the cellulose network) and de-polymerise it by hydrolysis and transelimination as well as by de-esterification reactions, which hydrolyses the ester bond between carboxyl and methyl groups of pectin (Ceci and Loranzo 1998; Thakur et al. 1997). The present investigation was undertaken to produce pectinase from the isolated fungal strain *Fusarium* sp. (PSTF1) in submerged fermentation for the conversion complex mango industrial waste into simpler degraded products (humus) as it has shown highest pectinolytic activity when compare with other fungal isolates.

## Materials and methods

### Sample collection

The processed waste (wet, dry and soil) of mango fruit processing industries was collected in sterile polythene covers from different areas around Chittoor, AP, India, and stored in refrigerator at 4 °C for further use. This waste (dried and powdered) used as carbon source/nutrition for isolates.

### Isolation and identification of fungal strains

The Fungal strains isolated by serial dilution of 1.0 g MIW. The pure cultures of isolates were made by Streak-plate method on Czepek dox’s agar media. The isolated fungal strains taxonomically identified by lacto phenol cotton blue method (Ref. Aneja Lab Manual 2004).

### Primary screening (screening test-I)

Modified Czapec dox’s broth used as production medium and screening has done on Czapec dox’s agar medium.

*Pectinase production medium (PPM)* This medium consists of part (A) and part (B). Part (A) contained (g/l): NaNO_3_, 2; KH_2_PO_4_, 1; KCl, 0.5; MgSO_4_ ·7H_2_O, 0.5; yeast extract, 1. These contents were dissolved in 40 ml distilled water. The pH was adjusted to pH 7.0 by NaOH (5 %, w/v). Part (B) contained (g/l): pectin, 5, dissolved in 10 ml of distilled water. The two parts (A) and (B) were mixed and sterilized. Then inoculated with spore suspension (0.1/25 ml) of fungal isolates and incubated at 30 °C for 96 h.

*Pectinase activity assay medium* Czepek dox’s Agar medium with 4 %pectin and 1 % Congo red (w/v) was used as assay medium. Plates of the same size poured with equal amounts of sterilized assay medium. Upon solidification three wells were made, each well inoculated with 0.1 ml of PPM (culture filtrate). These plates incubated at 30 °C for 5–7 days. Clearing zones of the medium directly observed on plates and taken as the criteria for determining the Pectinase productivity.

### Secondary screening (screening test-II)

In secondary screening, pectinase was produced in submerged fermentation using mango fruit processing industrial waste powder Basal medium as production medium (MIWP-BM).

*MIWP-basal medium* The basal medium (BM) was prepared according to Vincent method (Vincent JM 1970). It contained the following (g/l): NaNO_3_, 2; K_2_HPO_4_, 0.5; KCl, 0.5 and yeast extract 1 %. These were dissolved in citrate phosphate buffer at pH 7.0 and supplemented with MIW powder (4 % w/v) separately. Then pH of this medium was adjusted to 7.0 and sterilized at 121 °C. This medium was inoculated with spore suspension of *Fusarium* sp. (PSTF1) and incubated at 30 °C for 6 days. The pectinase productivity was assayed for every 24 h.

*Pectinase assay* The pectinase activity was assayed by DNS method (Miller and Gail Lorenz 1959). 0.5 ml of culture filtrate was used as pectinase; 0.5 ml of 1 % Pectin was used as substrate. One unit of enzyme activity is equal to the 1 µmol of reducing sugars released, measured in terms of D-Galacturonic acid, produced as a result of enzyme-substrate reaction.

#### Factors influencing the pectinase productivity

*Effect of temperature* The *Fusarium* sp. (PSTF1) grew on the MFIWP-Basal Medium by conducting experiments at different temperatures viz. 18, 28, 38, and 48 °C, for 6 days. Measurement of enzyme productivity was performed by di-nitro salicylic acid (DNS) method using spectrophotometer at 540 nm.

*Effect of pH* The pH of the production medium of different culture flasks were adjusted to 5, 6, 7, 8, by using 0.1 N NaOH and 0.1 N HCl. Then flasks were inoculated with spore suspension of *Fusarium* sp. (PSTF1) and incubated at 28 °C. Then the pectinase activity was measured for every 24 h using spectrophotometer at 540 nm.

*Effect of substrate concentration* Different concentrations of substrate [g/100 ml, (w/v)] 0.2, 0.4, 0.6, 0.8 were added to production medium, pH adjusted to 6.0. Then flasks were inoculated with spore suspension of *Fusarium* sp. (PSTF1) and incubated at 28 °C. Then the pectinase activity was measured for every 24 h using spectrophotometer at 540 nm.

*Effect of inoculum-size* The heavy spore suspension of the *Fusarium* sp. (PSTF1) was prepared by harvesting slants in 100 ml of sterile saline solution under aseptic conditions. The inoculum sizes (ml/100 ml) 0.2, 0.4, 0.6, 0.8 were added to flasks containing production medium (pH 6.0, with 0.6 g of substrate). Then inoculated flasks were incubated at 28 °C. Then the pectinase activity was measured for every 24 h using spectrophotometer at 540 nm.

*Effect of different incubation periods* Under the suitable culture conditions such as pH-6.0, substrate concentration (0.6 g) production medium was inoculated with 0.6 ml *Fusarium* sp. (PSTF1) spore suspension and incubated at 28 °C, respectively. The pectinase activity was measured every day at 2, 4, 8, and 16 h of time intervals at 540 nm.

*Effect of carbon source* Different external carbon sources were introduced into the production medium at an equimolecular amount located at 1 % (w/v) sucrose. Parallel experiment was made with no sugar as a control. The carbon sources, dextrose, fructose, and lactose and mannose and pectin were introduced at the level of 1 % (w/v). Under the above mentioned cultural conditions the culture flasks were inoculated and incubated for 100 h. Then the pectinase activity was measured for every 24 h using spectrophotometer at 540 nm.

*Effect of nitrogen source* Production medium was supplemented with different nitrogen sources at an equimolecular amount of nitrogen that present in sodium nitrate (0.2 %, w/v) in basal medium. The applied nitrogen sources ammonium oxalate, potassium nitrate, peptone, urea introduced as organic nitrogen source at the level of 1 % and the control was devoid from any nitrogen source. All experiments were carried out at above mentioned cultural conditions. Then the pectinase activity was measured for every 24 h using spectrophotometer at 540 nm.

*Effect of amino acids* The production medium was added at an equimolecular amount of nitrogen located in the best inorganic nitrogen source for the pectinase productivity. This experiment was controlled by performing of parallel one containing the original nitrogen source i.e., sodium nitrate and was devoid of any amino acid. The supplemented amino acids: alanine, glycine, phenyl alanine, and methionine. All experiments were carried out at above mentioned cultural conditions. Then the pectinase activity was measured for every 24 h using spectrophotometer at 540 nm.

*Effect of vitamins* Different vitamins are ascorbic acid, riboflavin, vitamin-B_6_ and vitamin-E added separately to flasks containing the pectinase production medium, while the control applied free from any vitamin. All experiments were carried out at above mentioned cultural conditions. Then the pectinase activity was measured for every 24 h using spectrophotometer at 540 nm.

## Results and discussion

### Sample collection

Thirty mango fruit processing industrial waste samples collected from ten different mango fruit processing industries. They were used as good source for the isolation of pectinase producing fungal strains. Earlier the wastes like Orange peel, Citrus peel and Potato peel were also used as a source for isolating pectinolytic microorganisms (Geetha et al. 2012).

### Isolation and identification of strains

Eight fungal strains isolated and pure cultured on Czapecdox’s agar slants. The isolated fungal strains identified as *Fusarium* sp. (PSTF1), *Lichtheimia* sp. (PSTF2), *Pseudolagarobasidium* sp. (PSTF3), *Pencillium* sp. (PSTF4), *Cladosporium* sp. (PSTF5), *Fusarium* sp. (PSTF6), *Fusarium* sp. (PSTF7), *Fusarium* sp. (PSTF8). Similarly fungal strains such as *Aspergillus niger*, *A. fumigates* and *A. nidulans*, *A. flavus* and *Penicillium chrysogenum* were isolated from agro and fruit processing industrial wastes (Reda et al. 2008; Attyia and Ashour 2002; Satvinder et al. 2004; Sudheer Kumar et al. 2010).

### Primary screening (screening test-I)

**Fig. 1:**
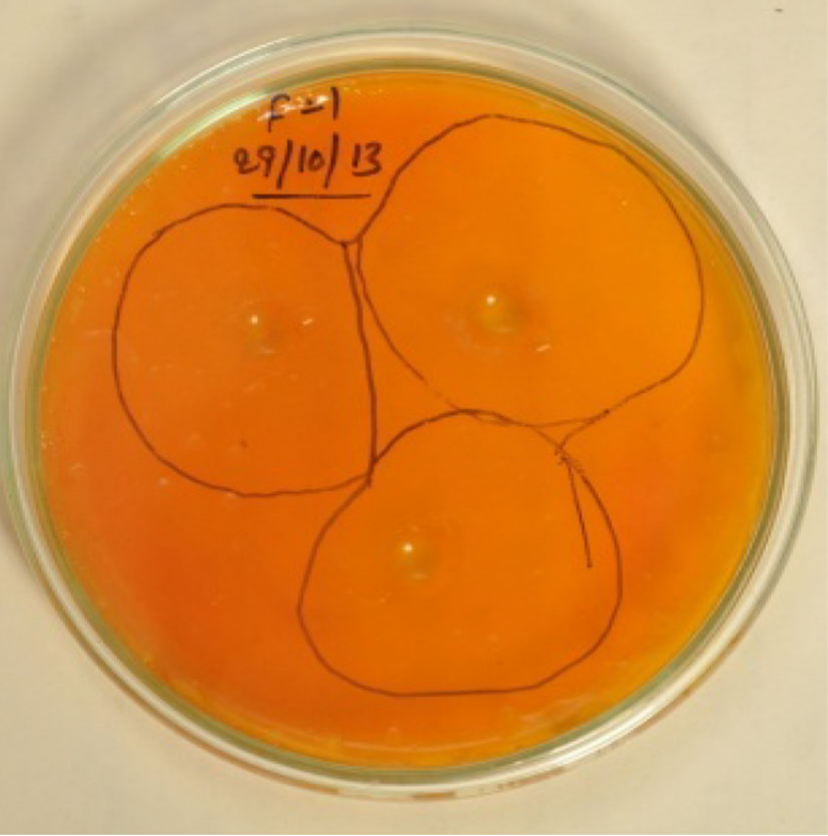
Showing pectin clear zones of *Fusarium* sp. (PSTF1)

All eight strains are having good pectinase productivity with PCZ values (Table 1). *Fusarium* sp. has given highest pectinase productivity, with 52 mm of PCZ value (Fig. 1). This PCZ value is higher than the earlier reported *Penicillium* sp., *Rhizopus* sp., *Neurospora* sp., *Mucor* sp. activities (Sandhya and Kurup 2013).

**Table 1:** Fungal isolates showing Pectinase productivity by clear zone (PCZ) in mm

S. no.	Isolate name	PCZ (mm)
1	*Fusarium* sp. (PSTF1)	52
2	*Lichtheimia* sp. (PSTF2)	26
3	*Pseudolagarobasidium* sp. (PSTF3)	22
4	*Pencillium* sp. (PSTF4)	15
5	*Cladosporium* sp. (PSTF5)	19
6	*Fusarium* sp. (PSTF6)	16
7	*Fusarium* sp. (PSTF7)	18
8	*Fusarium* sp. (PSTF8)	18

*mm* millimeter

#### Secondary screening (screening test-II)

##### Factors influencing the pectinase productivity

Pectinases are mainly used in the fruit processing industry to clarify the juice and increase the yields. In recent years a number of applications are being suggested and commercially applied, especially for pectinases (Kashyap et al. 2001). For this reason *Fusarium* sp. (PSTF1) had examined for its ability to utilize mango industrial waste as substrate for pectinase production under the influence of different factors. The physico-chemical characteristics of the fermentation medium such as temperature, pH, inoculum size, incubation time, carbon source, nitrogen source etc… played an important role pectinase production by *Fusarium* sp. (PSTF1).

**Figs. 2 and 3:**
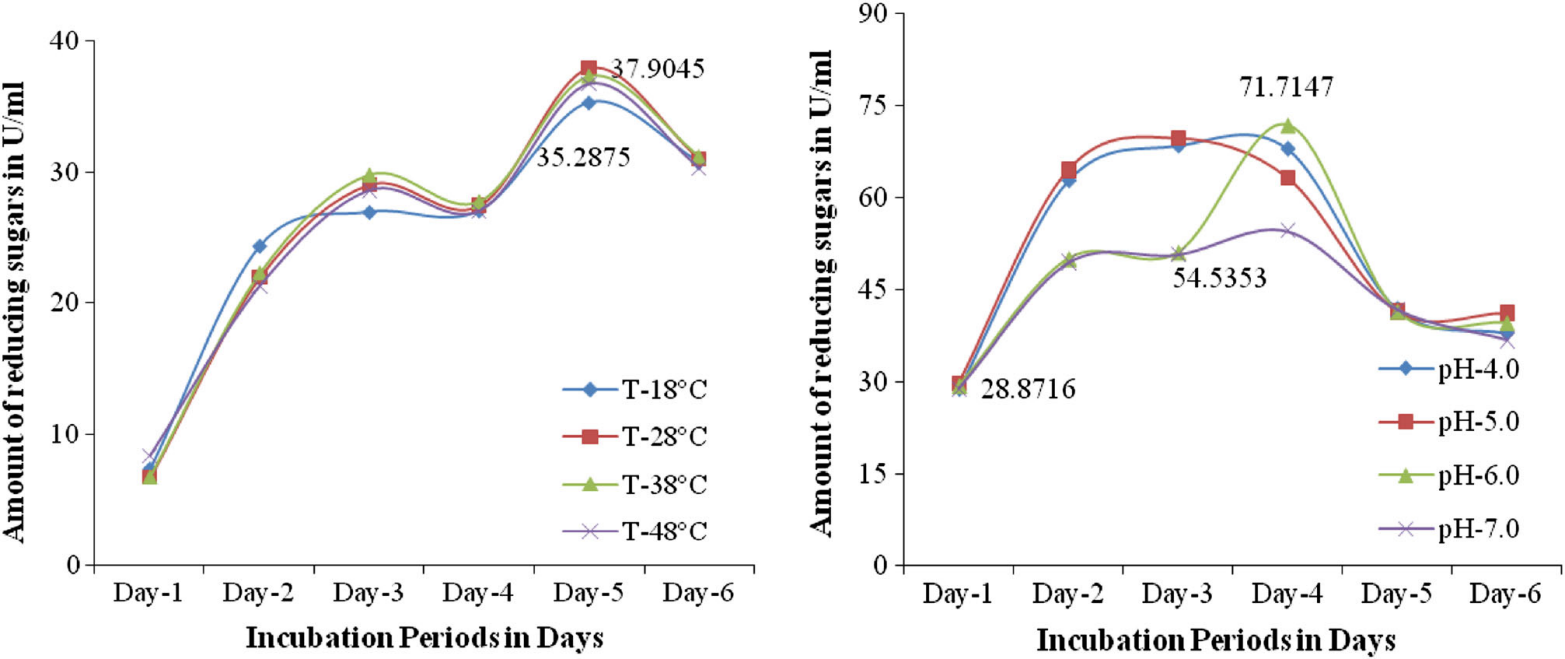
Effect of temperature and (°C) on the pectinase production by *Fusarium* sp. (PSTF1)

*Effect of temperature (°C)* The highest pectinase production (37.9045 U/ml) was observed at 28 °C. Upon increasing or decreasing the temperature the pectinase production was decreased (35.2875 U/ml) (Fig. 2). Temperature plays an important role on the growth of fungi; under the optimal temperature the growth rate of *Fusarium* sp. (PSTF1) was increased, as the growth rate increased the pectinase production was also increased. Patil et al. (2006) reported that the maximum amount pectinase was produced by *Aspergillus niger* DMF27 in submerged fermentation at optimal temperature 34 °C i.e., 12.6 U/ml. The maximum pectinase activity of *Fusarium* sp. (PSTF1) at 28 °C was higher than the activity of reported strain. This shows that the optimal temperature for high pectinase production by *Fusarium* sp. (PSTF1) is 28 °C.

*Effect of pH* The effect of pH on the pectinase production by *Fusarium* sp. (PSTF1) was determined. The pectinase production was ranged between 28.8716 and 71.7147 U/ml. The highest pectinase production of 71.7147 U/ml was observed at pH 6.0 and lowest pectinase production 54.5353 U/ml was observed at pH 7.0 (Fig. 3). This shows that the optimal pH for high pectinase production by *Fusarium* sp. (PSTF1) is 6.0.

**Figs. 4 and 5:**
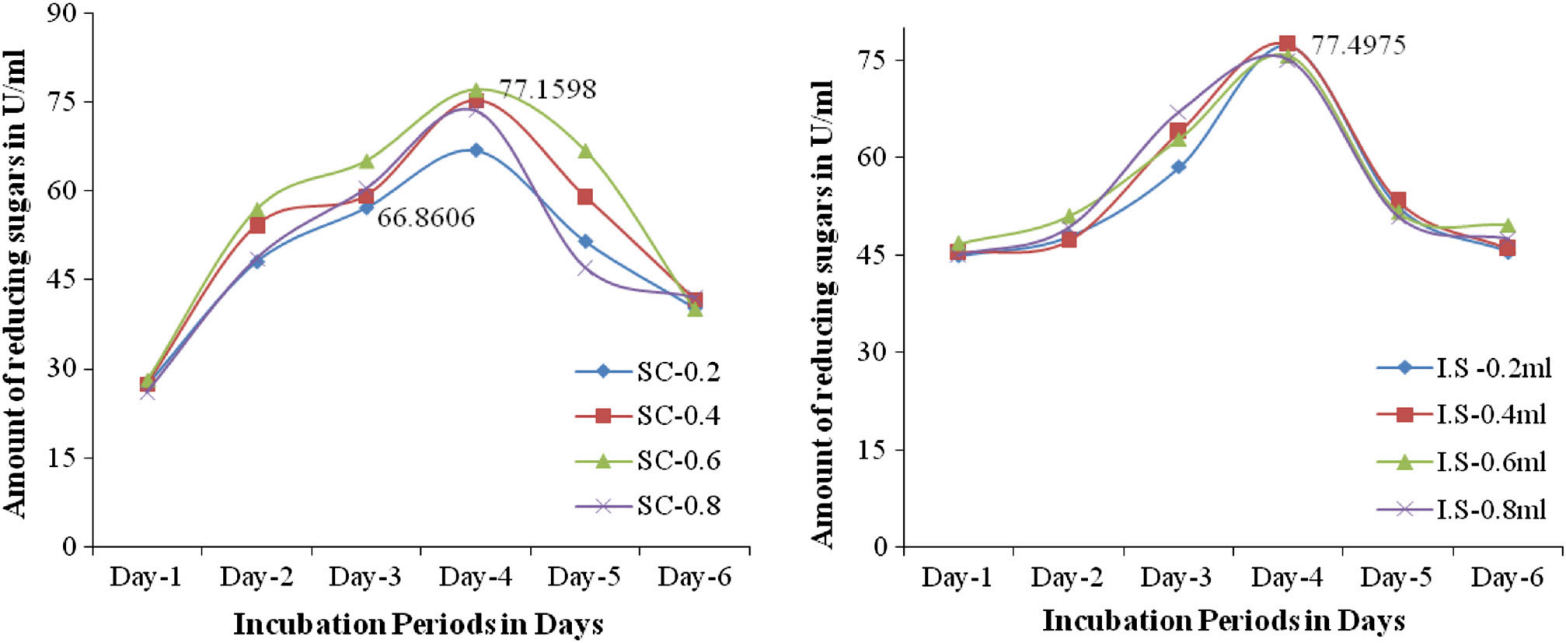
Effect of substrate concentration (g) and inoculum size (ml) on the pectinase production by *Fusarium* sp. (PSTF1)

*Effect of substrate concentration (g)* The Effect of Substrate concentration on pectinase production by *Fusarium* sp. (PSTF1) was determined at different substrate concentrations. The highest pectinase production (77.1598 U/ml) was observed with 0.6 g/100 ml of substrate (mango powder). Upon increasing or decreasing the substrate concentration the pectinase production by *Fusarium* sp. (PSTF1) was decreased to 66.8606 U/ml (Fig. 4). This shows that the suitable concentration is 0.6 g per 100 ml of production medium. Similarly, Palaniyappan et al. (2009) was used wheat flour and corn flour as substrates for the production of pectinase by *Aspergillus niger* MTCC 281. They found that the maximum activity of pectinase 5.16 U/ml (wheat flour) and 5.17 U/ml (corn flour) at 1 % concentration of substrate. The highest pectinase production of *Fusarium* sp. (PSTF1) using mango industrial waste as substrate was 14 times higher than the reported stain.

*Effect of inoculum size (ml)* The concentration of the inoculum and age of spores had a pronounced effect on pectinase production. The pectinase showed maximum activity (77.4975 U/ml) during the fourth day of incubation with 0.4 ml inoculum (Fig. 5). Obtained results suggested that the optimal inoculum concentration is 0.4/100 ml of culture medium and optimal age of spores is 4 days old for the production of pectinase by *Fusarium* sp. (PSTF1) in submerged fermentation. Similar results were found with pectinase production by *Aspergillus niger* MK-15 in submerged fermentation, the optimal inoculum concentration was 3 % (3/100 ml) spores and optimal age of spores was 3 days old (Kiro 2010). This showed that the low inoculum size of *Fusarium* sp. (PSTF1) yielded the pectinase activity better than the reported strain. Ibrahim et al. (2013) studied the effect of different inoculum sizes on pectinase production by *Aspergillus niger* HFD5A-1. Their results showed that the maximal production 1.64 U/ml at the inoculums size of 2 % (2/100 ml).

**Figs. 6 and 7:**
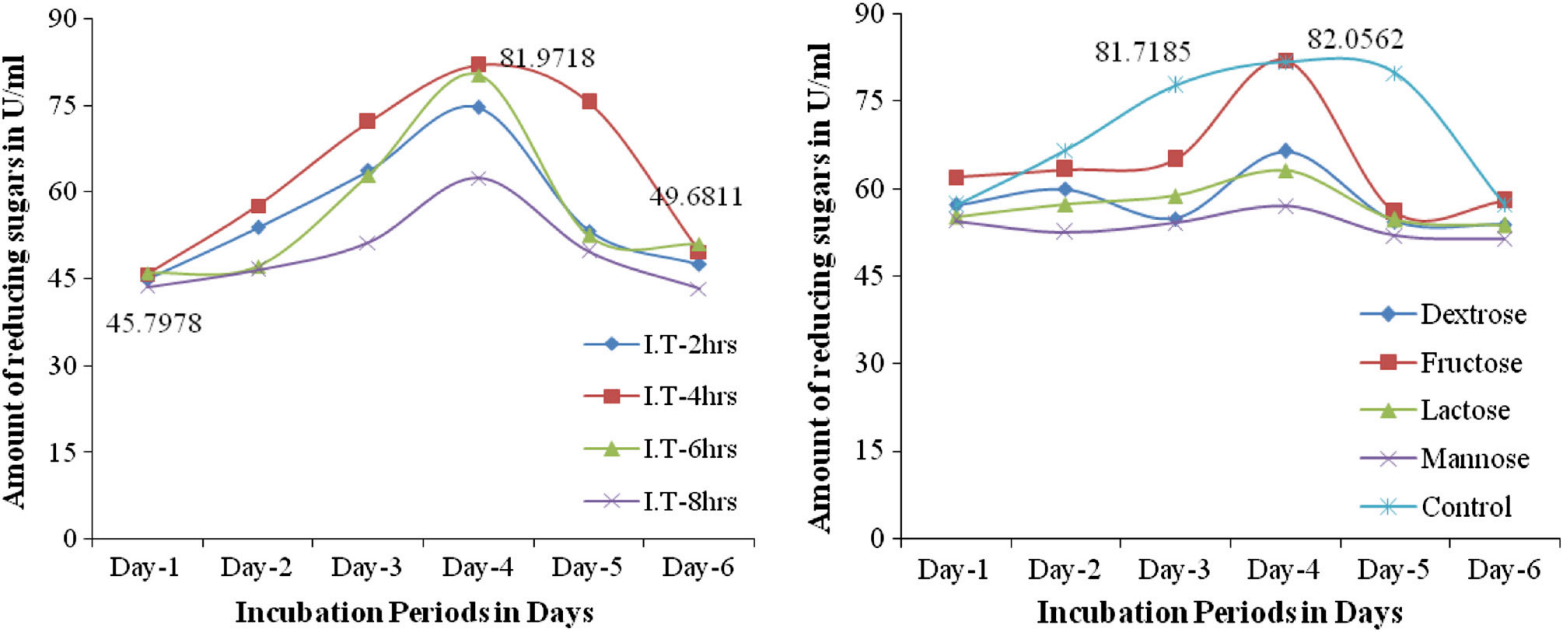
Effect of incubation periods (h) and carbon source (g) on the pectinase production by *Fusarium* sp. (PSTF1). *Dex* dextrose, *fru* fructose, *lac* lactose, *man* mannose, *con* control

*Effect of incubation periods (h)* The effect of different time intervals on pectinase production by *Fusarium* sp. (PSTF1) was determined. Initially, the pectinase production (45.7978 U/ml) was observed at 28 h of incubation, it was increased to 81.9718 U/ml at 100 h of incubation and then it was decreased to 49.6811 U/ml at 148 h (Fig. 6). This shows that the incubation time had prominent effect on pectinase production by *Fusarium* sp. (PSTF1) and the optimal incubation time for pectinase production is 100 h. The pectinase activity of *Fusarium* sp. (PSTF1) (81.9718 U/ml) under the standardized cultural conditions is very much higher than the activity of reference stains *Aspergillus niger* ATCC 9642 (30 U/ml) and *W23* (41 U/ml), *W43* (43 U/ml), and *D2* (*Penicillium* sp.) (45 U/ml) isolated from soil of a plum tree orchard and agro-industrial waste (Sunnotel and Nigam 2002; Zeni et al. 2011).

*Effect of carbon source (1* *%)* The effect of different carbon source on the pectinase production by *Fusarium* sp. (PSTF1) was determined. The highest pectinase production (82.0562 U/ml) was observed in presence of fructose (external carbon source) was higher than the pectinase production (81.7186 U/ml) of control (Fig. 7). The presence of other external carbon sources such as dextrose, lactose, and mannose were decreased the pectinase production than the control. This shows that the carbon source present in production medium had played an important role i.e., the increased concentration of carbon source decreased the growth of *Fusarium* sp. (PSTF1). So, the pectinase production was decreased. Ashfaq et al. (2012) reported that the presence of 1 % dextrose used as carbon source showed better result (2.73 U/ml). Similarly, Palaniyappan et al. (2009) found that the maximum pectinase activity of 5.17 and 5.84 U/ml with *Aspergillus niger* MTCC 281 using 1 % wheat flour and corn flour as substrates. Similarly, Rajiv Dhital et al. (2013) reported that the maximum pectinase production was observed on the yeast glucose pectin media enriched with pectin at the concentration of 1.5 % which was 21 U/ml by soil fungal isolate (MG1).

**Figs. 8 and 9:**
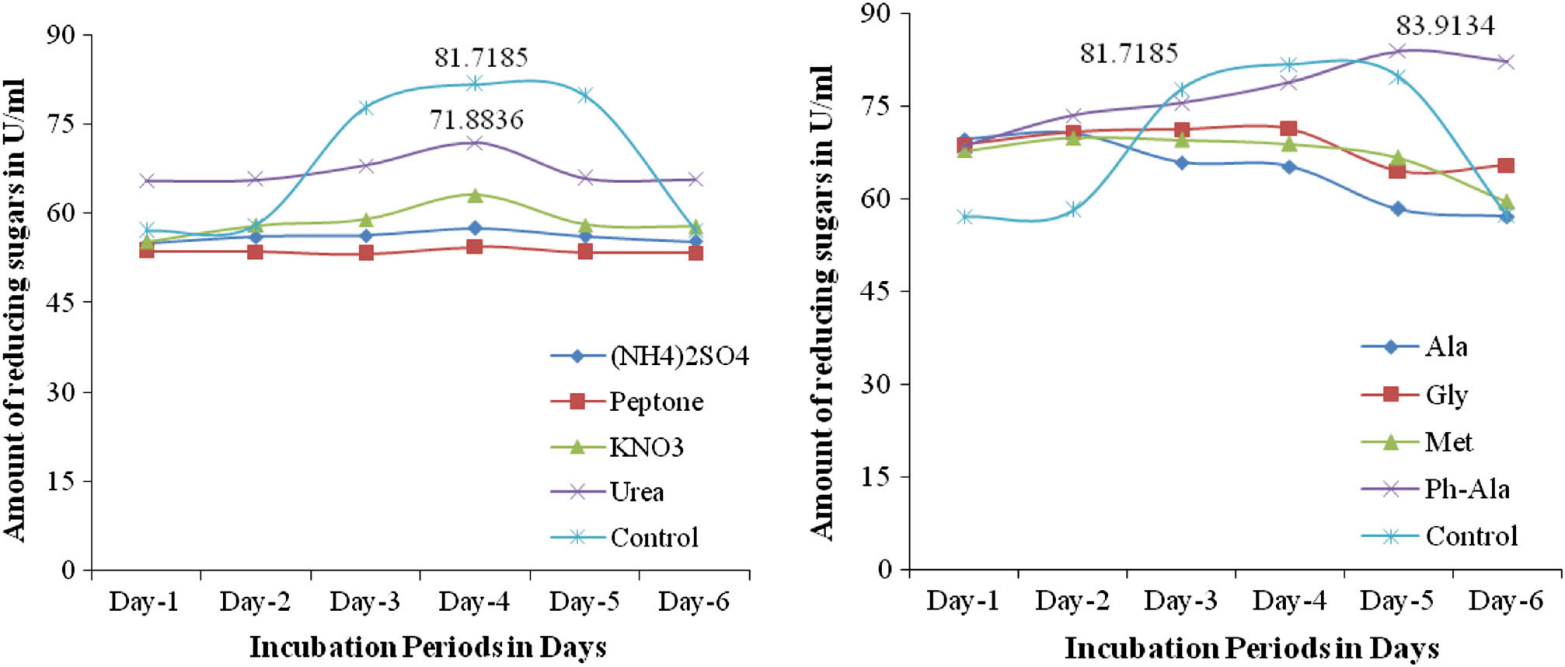
Effect of nitrogen source (1 %) and amino acids (1 %) on the pectinase production by *Fusarium* sp. (PSTF1)

*Effect of nitrogen source (1* *%)* The effect of different nitrogen sources on pectinase production by *Fusarium* sp. (PSTF1) was determined. The presence of external nitrogen source in the production medium decreased the pectinase production to 71.8836 U/ml while control showed high pectinase production (81.7185 U/ml) (Fig. 8). This showed that the increased concentration of nitrogen source in production medium was decreased pectinase production. The results suggested that there was no need of external nitrogen source for pectinase production by *Fusarium* sp. (PSTF1). But when compared with other nitrogen source in presence of urea highest pectinase activity was observed. Similarly, *Aspergillus niger* HFM-8 has given highest pectinase production in presence of external nitrogen source urea with citrus pectin (Ibrahim et al. 2013). Additional nutrients (other than substrate) in the growing media enhance the growth of fungi and growth related enzymes secretion. Addition of 0.1 % of peptone (% of total dry substrate) as a nitrogen source further enhanced the production of pectin lyase (Sidra et al. 2013).

*Effect of amino acids (1* *%)* The effect of amino acids (ala-alanine, gly-glysine, phe-phenyl alanine, met-methionine) on the pectinase production by *Fusarium* sp. (PSTF1) was determined. The presence of alanine, glysine, methionine in production medium increased the concentration nitrogen. So, the pectinase production was decreased. In presence of phenyl alanine pectinase production (83.9134 U/ml) was increased than the pectinase production (81.7185 U/ml) of control (Fig. 9). This showed that the phenyl alanine was the suitable amino acid for high production of pectinase by *Fusarium* sp. (PSTF1).

**Fig. 10:**
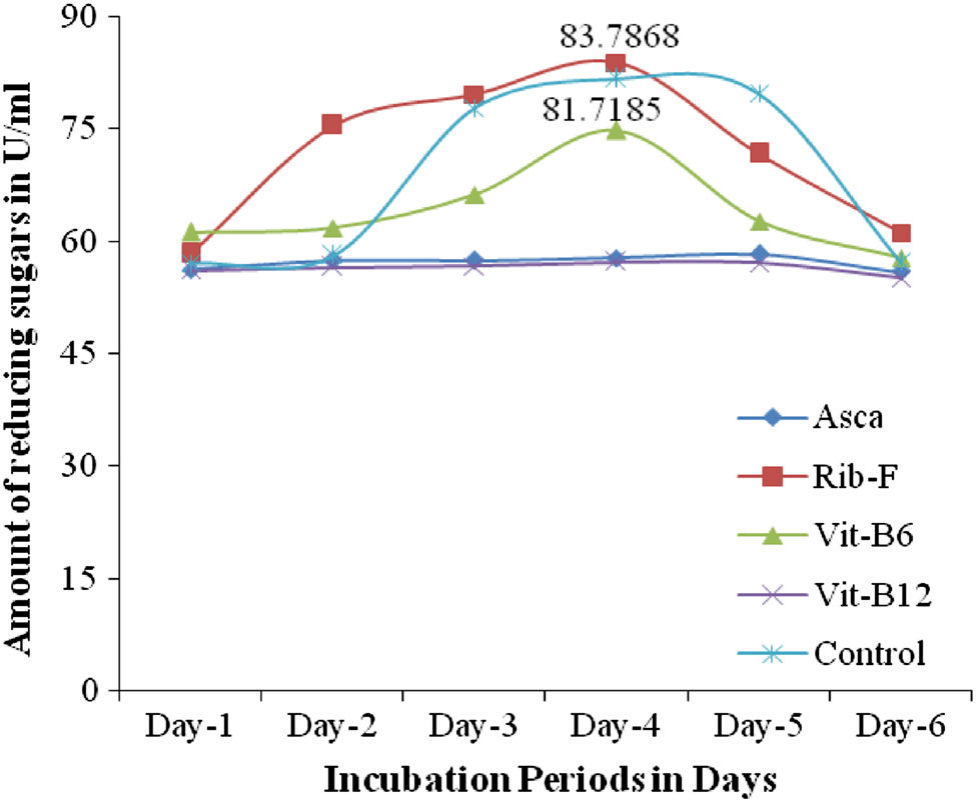
Effect of vitamins (1 %) on the pectinase production by *Fusarium* sp. (PSTF1)

*Effect of vitamins (1* *%)* The effect of vitamins on the pectinase production by *Fusarium* sp. (PSTF1) was determined. The presence of vitamins riboflavin and ascorbic acid had shown positive effect on pectinase production i.e., increased the pectinase activity to 83.7868 U/ml while the control had shown 81.7185 U/ml (Fig. 10). The other vitamins had not shown significant effect on pectinase production. The results are suggesting that the riboflavin was the suitable media component for high production of pectinase by *Fusarium* sp. (PSTF1). The presence of external carbon source (Fructose) and added vitamins (Riboflavin) in the production medium increased the pectinase productivity to 82.647 and 83.786 U/ml. Suresh and Viruthagiri (2010) reported that the maximum pectinase yield from *Aspergillus niger* was observed with temperature-40 °C, pH-5.0, mixed substrates 10 g (90 % of wheat bran and 10 % of sugarcane bagasse), fermentation period of 96 h. This showed that the chemical composition of the medium will also played an important role in pectinase production from *Fusarium* sp. (PSTF1).

## Conclusion

The results of present study stated that Fusarium sp. (PSTF1) is a better pectinase producer than aforementioned strains, based on the fact that the PCZ of PSTF1 is twice as that of the second most effective strain. The pectinase activity of 83.786 U/ml observed under suitable submerged fermentation conditions by *Fusarium* sp. (PSTF1) is higher than the earlier reported strains. In addition to these, some additional properties like external carbon source (Fructose) and added vitamins (Riboflavin), low substrate concentration, less incubation time for pectinase production indicating the potential of *Fusarium* sp. (PSTF1) to be used at commercial level in industries. An overview of the results obtained show that simple submerged fermentation of mango fruit processing industrial waste was suitable to produce low cost, high value product i.e., Pectinase by *Fusarium* sp. (PSTF1). The environmental pollution will also reduced by using mango fruit processing industrial waste as whole and sole carbon source for the production of pectinase. The Pectinase enzyme itself can also find a number of applications in the mango processing industry.
